# Pharmacists' viewpoint towards their professional role in healthcare system: a survey of hospital settings of Pakistan

**DOI:** 10.1186/s12913-020-05459-0

**Published:** 2020-07-02

**Authors:** Nabeel Khan, Ken McGarry, Atta Abbas Naqvi, Muhammad Shahid Iqbal, Zaki Haider

**Affiliations:** 1 School of Pharmacy and Pharmaceutical Sciences, University of Sunderland, Sunderland, UK; 2 Faculty of Technology, School of Computer Science, University of Sunderland, Sunderland, UK; 3 Department of Pharmacy Practice, College of Clinical Pharmacy, Imam Abdulrahman Bin Faisal University, Dammam, Saudi Arabia; 4 Department of Clinical Pharmacy, College of Pharmacy, Prince Sattam bin Abdulaziz University, Alkharj, Saudi Arabia; 5 Department of Health and Science Management, Bahria University, Karachi, Pakistan

**Keywords:** Pharmacists, Clinical pharmacists, Hospital pharmacy service, Clinical pharmacy service, Pakistan

## Abstract

**Background:**

Pharmacy service is an essential part of a healthcare system. The profession of pharmacy is well recognized and is practiced to its full potential in developed countries however, it is underutilized in developing countries such as Pakistan. The recognition of pharmacist’s role as healthcare professional is limited. This study aimed to document pharmacists’ attitude towards their role in Pakistan’s healthcare system, their experience with doctors and their perceptions towards involvement in medicines management.

**Methods:**

A 4-month cross-sectional survey (Jan – Apr 18) was conducted targeting pharmacists practising in 26 tertiary care hospitals across Pakistan using a developed and validated questionnaire in both Urdu/English languages. Chi square (χ^2^) test was used to report any associations between independent variables, i.e., education, type of hospital and work experience and, dependent variables, i.e., pharmacists’ attitudes, experience, and perception. A *p*-value of ≤0.01 with value of Cramer’s V ≥ 0.3 was considered cut-off for establishing statistical significance. The study was approved by ethical committee and local hospital committees.

**Results:**

Three hundred ninety-six questionnaires were returned out of 500, i.e., response rate = 87.9%. Most participants (92.2%) interacted with doctors at least once daily. Most interactions were related to drug availability inquiry (72.5%). Most pharmacists (91.4%) mentioned that pharmacy duties are mostly clinical in nature. 93.4% of the respondents indicated that pharmacists are reliable source of information regarding general medicines. Furthermore, 87.4% reasoned inadequate training for not being able to discuss issues of clinical nature with doctors.

**Conclusion:**

Pharmacists were willing to perform their duties and provide healthcare benefits to patients however, they seemed sceptical of advanced clinical pharmacy roles such as intervening in prescriptions and medication therapy, consultations and prescribing. There is a need to increase awareness regarding pharmacist’s role. Therefore, it would be helpful if trainings and seminars are conducted on the importance of clinical pharmacy to improve the pharmacy services in Pakistan’s healthcare system.

## Introduction

The profession of pharmacy has evolved from a product-focused practice to a patient-oriented one [1]. In general, the primary duties of the pharmacist include providing drug information, medicines management, preparation and dispensing of medicines, counselling of patients, and formulating pharmaceutical care plan for patients [2]. Pharmaceutical care plan is an individualized service provided by pharmacist that aims to improve the quality of patient’s health [3, 4]. Pharmaceutical care involves a collaborative relationship between the pharmacist and the physicians to improve the health status of patients [5]. It was envisioned that in future, pharmacists would be greatly involved in clinical and administrative roles and, their traditional roles of pill counting, packaging, and dispensing would be performed by technicians and trainees [6]. Literature suggests that pharmaceutical care practice has a substantial positive effect on healthcare and disease management in developed countries [7, 8]. However, the situation is different in developing countries as the application of pharmaceutical care is hindered due to time constraints, lack of standard reimbursement, less access to patients’ records, poor communication among healthcare professionals, insufficient number of qualified pharmacists and absence of policies [8].

A pre-requisite to establishing effective pharmaceutical care services would be the greater involvement of hospital pharmacist as a member of allied health team with increased interaction with other healthcare professionals. Effective implementation of this service requires an understanding of a hospital pharmacist’s perception towards the concept of pharmaceutical care, their role in direct patient care and, the extent and level of interaction with other healthcare professionals [9]. The traditional role of pharmacist has always been procurement and inventory management of medicines along with ensuring their safety and efficacy [10]. However, with the introduction of ‘Pharmaceutical care’ concept by Hepler and Strand during the 90s, the tradition role of pharmacist has transcended from medicine provider to a patient care provider [11]. During the last few decades, pharmacists have practiced pharmaceutical care to improve patients’ treatment outcomes and maximize the benefits of medication therapy to patients [5, 12].

According to the World Health Organization (WHO), pharmacists are expected to advise allied health team regarding medication therapy management in patient care and must have specialized knowledge and skills needed to execute clinical pharmacy services [13, 14]. In Pakistan, the health authorities have resolved to introduce the pharmaceutical care services within the healthcare system to improve patient’s quality of life [15]. However, pharmaceutical care model is a novel concept within pharmacy practice domain and is not clear to a majority of health professionals and public [16]. Besides, there are few pharmacists with knowledge of pharmaceutical care and clinical pharmacy services in healthcare system. In addition, clinical guidelines are either not updated or not available, that could highlight pharmacist’s role in patient care. These are few notable determinants of an enhanced clinical role of pharmacist in Pakistan’s healthcare system [9, 17]. In addition, there is an acute shortage of pharmacists in all healthcare sectors of the country. Hospital pharmacists are involved to a greater extent in traditional pharmacy services and administrative activities. Furthermore, it has been observed that pharmacy graduates in Pakistan preferred to join pharmaceutical marketing and sales jobs [17–19].

Pharmacy curriculum in Pakistan has traditionally been inclined towards drug manufacturing and dispensing. During the last decade, the healthcare policymakers envisioned a clinical role of pharmacist and since then, the pharmacy curriculum has been modified to incorporate courses related to clinical pharmacy practice [20–22]. Moreover, the current pharmacy practice model in Pakistan requires modifications concerning collaborative practice supported by evidence and should have a clear perspective in its application and conceptualisation [23, 24]. As this paradigm is novel in Pakistan, it requires more planning for future perspectives suitable to the country’s health policies. At the same time, it is imperative to find out what pharmacists perceive about pharmaceutical care service and their supposed engagement with the other medical staff, to understand the determinants that hinder their clinical role in Pakistani healthcare settings.

Available literature highlights that interprofessional relationship between doctors and pharmacists was not satisfactory in the past. One of the reasons was education and training of pharmacists [20–24]. Azhar and colleagues identified that the primary reasons for doctors’ low expectations with pharmacists were deficiency in knowledge of therapeutics and inadequate clinical pharmacy training [23]. In addition, Khan and colleagues highlighted that workplace and experience of healthcare professionals could influence views towards pharmacists and their services [24]. Hence, having pharmacy education that includes clinical courses is vital in shaping the perception and expectations of pharmacists regarding healthcare system of Pakistan.

In the past, adequate attention was not paid to update pharmacy curriculum and thus, it could not contribute significantly in achieving the healthcare objectives. The 4-year Bachelor of Pharmacy (BPharm) degree was upgraded to a 5-year Doctor of Pharmacy (PharmD) program by the Higher Education Commission (HEC) of Pakistan in 2004. The course introduces an intensive knowledge of clinical aspects of pharmacy. The updated program had courses that provided the knowledge of; clinical and social aspects of pharmacy, such as drug abuse, geriatric pharmacy, patient counselling, patient compliance, research methods, and evidence-based medicine, that have been largely ignored previously [9, 21, 22].

The health authorities of Pakistan should implement pharmaceutical care practices in the health system of the country, to promote safe use of medicines and improve patients’ quality of life [23, 24]. The present study was conducted to evaluate the acceptability of pharmacists as a patient care provider, their interaction with the doctors and, their perception about performing medicines management services. Thus, the study evaluated pharmacists’ viewpoint regarding their professional role in the healthcare system of Pakistan.

## Methodology

### Study objective

The primary objective of this study was to document attitude of pharmacists towards their role in Pakistan’s healthcare system, their experience with doctors and their perceptions towards involvement in medicines management.

### Study design, duration, and venues

**Fig. 1:**
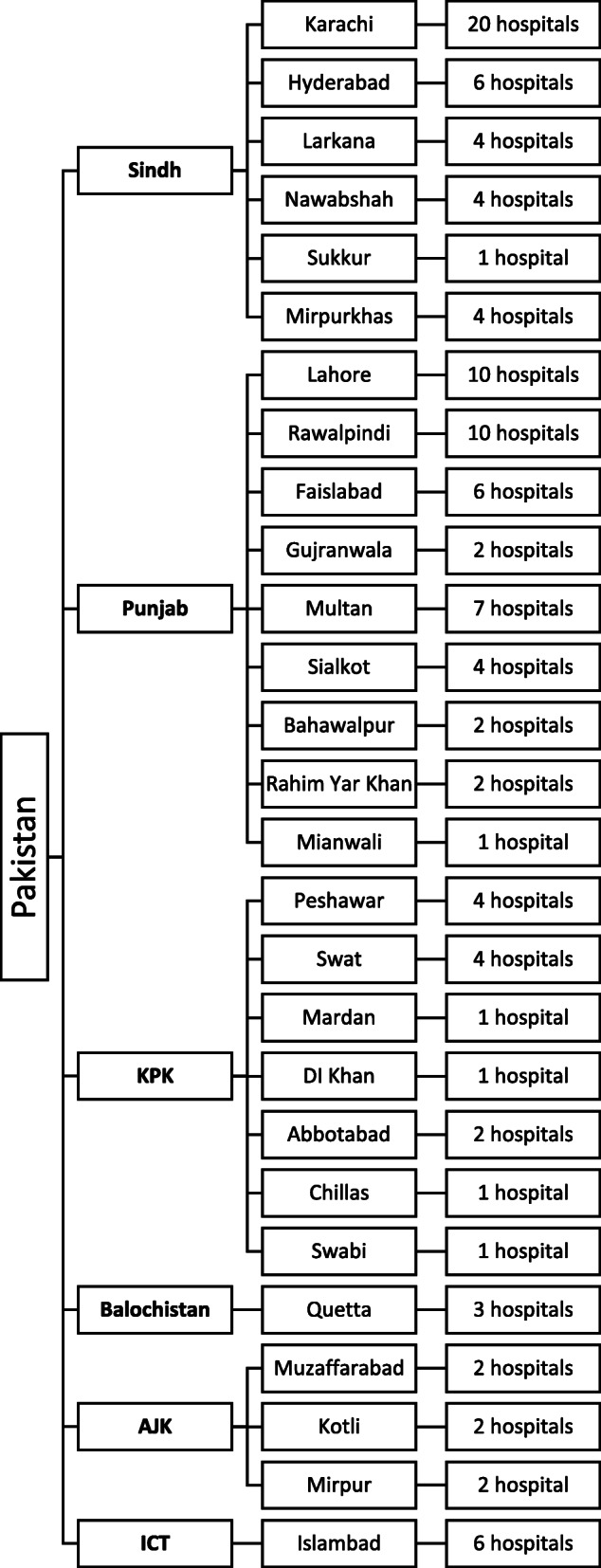
Study venues

This study was a cross-sectional survey that was conducted for a period of 4 months, i.e., from January 2018 up to April 2018. Pakistan is located in South Asia and hosts a population over 220 million. The healthcare system of Pakistan consists of state funded and private sector healthcare facilities [25]. There are 968 state funded hospitals across 8 administrative units of the country. Since the state funded healthcare structure is inadequate to fulfil healthcare needs of the population, most of Pakistanis utilize private sector hospitals as well [21]. The study was conducted in 122 tertiry care hospitals in 27 cities of Pakistan that were in 6 administrative units of the country (Fig. 1).

### Target population and eligibility criteria

The target population was pharmacists working in hospital settings in several cities of Pakistan. The eligibility criteria of pharmacists were adopted from Naqvi et al., i.e., licensed pharmacists who were currently working in healthcare settings of Pakistan for at least 1 year [14, 23]**.** All licensed pharmacists working in the pharmaceutical industry, academia and community pharmacies were excluded. They were excluded from study since they were not working in the healthcare settings and may have different perceptions that may not be representative.

### Sampling strategy and sample size calculation

Data collection was conducted using convenience sampling technique due to the unavailability of a database that could highlight the exact number of pharmacists practising in various hospitals across Pakistan. The study sample size was estimated using an online sample size calculator (RAOSOFT) [26]. As per the country’s pharmaceutical data obtained from WHO 2010 report, there were roughly 10,000 licensed pharmacists working in all settings of Pakistan [25]. This figure was considered as target population. A 5% margin of error and 95% confidence level was set. The required sample size was 370. A 2% drop-out rate (*N* = 8) was added to yield the final number of 378 pharmacists.

### Research instrument development and validation

The questionnaire was formulated for this study with help from previous literature by a panel of experts including two academicians and two clinical pharmacists [7, 23, 24]. There were four sections in the questionnaire that included demographic information about participants, the interactions of pharmacists with doctors, the reasons for their interactions and, the perception of pharmacists regarding their professional role in Pakistan’s healthcare settings. Apart from demographic questions, it included questions related to professional acceptance of pharmacists in the healthcare system, pharmacists’ experience of working with doctors and, the involvement of pharmacists in medicines management. The final questionnaire contained a total of 45 items and it took about 25 min on an average to fill in the response. The options for questions except for demographics were dichotomous, i.e., in yes/no format. The questionnaire was formulated in both English and Urdu languages. The English version of questionnaire is available as a supplementary file (additional file 1: Questionnaire).

The questionnaire was subjected to content validation by a panel of experts that included three academicians, two clinical pharmacists and three pharmacists. Each member of the panel reviewed the questions and indicated them as essential/non-essential. Content validation was conducted using the methodology described by Lawshe and Rungtusanatham [27, 28]. The content validity index was reported at 0.81 which was greater than the cut-off value 0.75 required for establishing validity [27]. The reliability of questionnaire was estimated using Cronbach alpha (α). The overall reliability for all items (*N* = 45) was reported at 0.889. The intraclass correlation coefficient was 0.889 (95% CI: 0.873–0.904). The reliability for the section of professional acceptance of pharmacists in the healthcare system that contained 17 items was 0.908. The intraclass correlation coefficient was 0.908 (95% CI: 0.894–0.920). Besides, the reliability for the section of pharmacists’ experience of working with doctors that had 15 items, was 0.861 and intraclass correlation coefficient was 0.861 (95% CI: 0.840–0.880). Moreover, the reliability for the section of involvement of pharmacists in medicines management that had 13 items, was 0.831 and intraclass correlation coefficient was 0.830 (95% CI: 0.810–0.850). All were in acceptable range [29, 30].

### Data collection

The questionnaire was delivered as hardcopy by hand and, were either completed at the same date or, collected later as indicated by the respondent. Prior to handing the questionnaire the participants were briefed about study purpose.

### Data analyses

The data were analysed using statistical software, (SPSS, Chicago, IL, USA, version 24.0). The results were reported, as sample count (N) and frequency (%). Cross tabulation and chi square (χ^2^) test was applied to assess the association between the independent variables (education, type of hospital and experience of pharmacists) and dependent variables (attitude, experiences of pharmacists with doctors and involvement of pharmacists in medicines management in the hospitals). The cut-off for statistical significance was *p*-value ≤0.01 with value for Cramer’s V ≥ 0.3. A *p*-value of ≤0.01 was set to reduce the likelihood type 1 errors [31]. The Cramer’s V indicated the strength of association and the value ranged from 0 to 1 with latter indicating a strong association. It was therefore selected as one of the cut-off criteria [32].

### Consent and ethics approval

All those who agreed to participate had to provide their consent before data collection. The study was approved by the Research Ethics Committee of the University (Reference Number 00286). In addition, approval letters from the local hospitals were also obtained before data collection.

## Results

Of total 500 pharmacists approached, 396 questionnaires were completed giving a response rate of 79.2%, the results of which were then taken forward for analysis. Most respondents were male (64.1%) and had Pharm. D (Doctor of Pharmacy) degree (74.5%). Almost equal proportions of respondents worked in state-funded (50.3%) and private hospitals (49.7%) respectively. As per the job titles of respondents, most respondents were working in capacity of pharmacist (91.7%). Among all respondents, almost 60% had experience of 5–10 years. Majority (66.9%) were associated with in-patient pharmacy. Almost all (99.2%) respondents were registered with Pakistan Pharmacy Council. The demographic information data are tabulated in Table 1.

**Table 1:** Participants’ information (*N* = 396)

Characteristics	N (%)
**Gender**	
Male	254 (64.1%)
Female	142 (35.9%)
**Professional Education**	
Bachelor of Pharmacy/ Master of Pharmacy	87 (22%)
Doctor of Pharmacy (Pharm.D)	295 (74.5%)
Overseas Qualification	14 (3.5%)
**Place of Work**	
State Funded Hospital	199 (50.3%)
Private Hospital	197 (49.7%)
**Current Job Title**	
Pharmacist	363 (91.7%)
Senior Pharmacist	26 (6.6%)
Chief Pharmacist	7 (1.8%)
**Years of Experience**	
Less than 5 years	68 (17.2%)
5–10 years	236 (59.6%)
More than 10 years	92 (23.2%)
**Area of Practice**	
Inpatient	265 (66.9%)
Outpatient	105 (26.5%)
Oncology pharmacy	26 (6.6%)
**State of Practice**	
Sindh	143 (36.1%)
Punjab	175 (44.2%)
Baluchistan	9 (2.3%)
KPK	44 (11.1%)
Capital Territory	17 (4.3%)
AJK	8 (2.0%)
**Are you registered with Pharmacy Council?**	
Yes	393 (99.2%)
In process	3 (0.8%)

*KPK* Khyber Pakhtunkhwa, *AJK* Azad Jammu and Kashmir

Upon eliciting opinion of pharmacists as to how they describe the pharmacy job, most of them (*N* = 362, 91.4%) mentioned that pharmacy duties are mostly clinical. However, a small number of pharmacists (*N* = 34, 8.6%) mentioned the duties as technical. In response to the question of how they would describe pharmacy as an occupation, most pharmacists (*N* = 351, 88.6%) mentioned it as a professional occupation while some pharmacists (*N* = 20, 5.1%) believed it to be a business profession. Similar number of pharmacists (*N* = 25, 6.3%) mentioned pharmacy job as both professional and business occupation.

Most participants (*N* = 365, 92.2%) reported that they interacted with doctors daily while some pharmacists (*N* = 18, 4.5%) reported that their interactions with doctors were on a weekly basis. Few pharmacists (*N* = 13, 3.3%) reported that they rarely interacted with doctors during their duty hours. In response to the question regarding most common reasons for these interactions, most pharmacists (*N* = 287, 72.5%) mentioned drug availability queries while some (*N* = 39, 9.8%) mentioned queries regarding drug alternatives. A small number of pharmacists (*N* = 29, 7.3%) mentioned queries related to drug interactions while a similar number of participants (*N* = 25, 6.3%) highlighted queries regarding dosage. Few pharmacists (*N* = 16, 4%) mentioned queries related to adverse drug reactions as one of the most common reasons for interactions. The data regarding pharmacists’ attitude towards their role in the healthcare system and their experience with doctors are tabulated in Tables 2 and 3. In addition, data related to the perception of pharmacists about their involvement in medicines management are tabulated in Table 4. All items were cross tabulated with three independent variables namely level of education of doctors, the nature of hospitals, and work experience of pharmacist.

**Table 2:** Attitude of pharmacist regarding their role in the healthcare system (*N* = 396)

Roles	Education	Agree	Disagree	***P***-Value	Type	Agree	Disagree	***P***-Value	Experience	Agree	Disagree	***P***-Value
To educate patients and carers about the safe and appropriate use of medicines	B/M Pharm	71 (17.9%)	16 (4%)	0.000*	State funded	172 (43.4%)	27 (6.8%)	0.035	< 5 years	65 (16.4%)	3 (0.8%)	0.001
	Pharm.D	275 (69.4%)	20 (5.1%)		Private	183 (46.2%)	14 (3.5%)		5–10 years	217 (54.8%)	19 (4.8%)	
	Overseas	9 (2.3%)	5 (1.3%)		–	–	–		> 10 years	73 (18.4%)	19 (4.8%)	
To monitor and report patients’ response to drug therapy	B/M Pharm	74 (18.7%)	13 (3.3%)	0.002	State funded	176 (44.4%)	23 (5.8%)	0.086	< 5 years	64 (16.2%)	4 (1.0%)	0.022
	Pharm.D	276 (69.7%)	19 (4.8%)		Private	184 (46.5%)	13 (3.3%)		5–10 years	219 (55.3%)	17 (4.3%)	
	Overseas	10 (2.5%)	4 (1.0%)		–	–	–		> 10 years	77 (19.4%)	15 (3.8%)	
To be available for clinician consultation during ward rounds	B/M Pharm	71 (17.9%)	16 (4.0%)	0.031	State funded	169 (42.7%)	30 (7.6%)	0.031	< 5 years	65 (16.4%)	3 (0.8%)	0.009
	Pharm.D	268 (67.1%)	27 (6.8%)		Private	181 (45.7%)	16 (4%)		5–10 years	211 (53.3%)	25 (6.3%)	
	Overseas	11 (2.8%)	3 (0.8%)		–	–	–		> 10 years	74 (18.7%)	18 (4.5%)	
To communicate or liaise with other healthcare professionals delivering patient care to facilitate positive health outcomes	B/M Pharm	78 (19.7%)	9 (2.3%)	0.005	State funded	193 (48.7%)	6 (1.5%)	0.000*	< 5 years	67 (16.9%)	1 (0.3%)	0.031
	Pharm.D	269 (67.9%)	26 (6.6%)		Private	163 (41.2%)	34 (8.6%)		5–10 years	209 (52.8%)	27 (6.8%)	
	Overseas	9 (2.3%)	5 (1.3%)		–	–	–		> 10 years	80 (20.2%)	12 (3.0%)	
To collaborate with other healthcare professionals as part of a multidisciplinary team	B/M Pharm	71 (17.9%)	16 (4.0%)	0.001	State funded	172 (43.4%)	27 (6.8%)	0.293	< 5 years	65 (16.4%)	3 (0.8%)	0.001
	Pharm.D	269 (67.9%)	26 (5.5%)		Private	177 (44.7%)	20 (5.1%)		5–10 years	213 (53.8%)	23 (5.8%)	
	Overseas	9 (2.3%)	5 (1.3%)		–	–	–		> 10 years	71 (17.9%)	21 (5.3%)	
To provide advice to patients about their medication/s and/or health conditions	B/M Pharm	71 (17.9%)	16 (4.0%)	0.000*	State funded	175 (44.2%)	24 (6.1%)	0.083	< 5 years	64 (16.2%)	4 (1.0%)	0.000*
	Pharm.D	278 (70.2%)	17 (4.3%)		Private	184 (46.5%)	13 (3.3%)		5–10 years	222 (56.1%)	14 (3.5%)	
	Overseas	10 (2.5%)	4 (1.0%)		–	–	–		> 10 years	73 (18.4%)	19 (4.8%)	
To dispense and check supply of medicines to patient (counting pills, labelling, and accuracy checking)	B/M Pharm	67 (16.9%)	20 (5.1%)	0.036	State funded	161 (40.7%)	38 (9.6%)	0.018	< 5 years	63 (15.9%)	5 (1.3%)	0.021
	Pharm.D	259 (65.4%)	36 (9.1%)		Private	176 (44.4%)	21 (5.3%)		5–10 years	203 (51.3%)	33 (8.3%)	
	Overseas	11 (2.8%)	3 (0.8%)		–	–	–		> 10 years	71 (17.9%)	21 (5.3%)	
To provide a “closed shop” service: receiving prescriptions from a practitioner and dispense medicine to a patient only	B/M Pharm	11 (2.8%)	76 (19.2%)	0.001	State funded	54 (13.6%)	145 (36.6%)	0.000	< 5 years	16 (4.0%)	52 (13.1%)	0.623
	Pharm.D	59 (14.9%)	236 (59.6%)		Private	24 (6.1%)	173 (43.7%)		5–10 years	46 (11.6%)	190 (48.0%)	
	Overseas	8 (2.0%)	6 (1.5%)		–	–	–		> 10 years	16 (4.0%)	76 (19.2%)	
To check that prescriptions are written for the correct dose for the patient	B/M Pharm	60 (15.2%)	27 (6.8%)	0.000*	State funded	152 (38.4%)	47 (11.9%)	0.955	< 5 years	52 (13.1%)	16 (4.0%)	0.014
	Pharm.D	239 (60.4%)	56 (14.1%)		Private	150 (37.9%)	47 (11.9%)		5–10 years	190 (48.0%)	46 (11.6%)	
	Overseas	3 (0.8%)	11 (2.8%)		–	–	–		> 10 years	60 (15.2%)	32 (8.1%)	
To check that prescriptions do not have any drug-drug interactions	B/M Pharm	55 (13.9%)	32 (8.1%)	0.017	State funded	156 (39.4%)	43 (10.9%)	0.093	< 5 years	61 (15.4%)	7 (1.8%)	0.004
	Pharm.D	231 (58.3%)	64 (16.2%)		Private	140 (35.4%)	57 (14.4%)		5–10 years	173 (43.7%)	63 (15.9%)	
	Overseas	10 (2.5%)	4 (1.0%)		–	–	–		> 10 years	62 (15.7%)	30 (7.6%)	
To check that a prescription is not contraindicated for the patient	B/M Pharm	67 (16.9%)	20 (5.1%)	0.036	State funded	161 (40.7%)	38 (9.6%)	0.018	< 5 years	63 (15.9%)	5 (1.3%)	0.021
	Pharm.D	259 (65.4%)	36 (9.1%)		Private	176 (44.4%)	21 (5.3%)		5–10 years	203 (51.3%)	33 (8.3%)	
	Overseas	11 (2.8%)	3 (0.8%)		–	–	–		> 10 years	71 (17.9%)	21 (5.3%)	
To advise clinicians and others about the cost-effectiveness of medicines	B/M Pharm	74 (18.7%)	13 (3.3%)	0.002	State funded	176 (44.4%)	23 (5.8%)	0.086	< 5 years	64 (16.2%)	4 (1.0%)	0.022
	Pharm.D	276 (69.7%)	19 (4.8%)		Private	184 (46.5%)	13 (3.3%)		5–10 years	219 (55.3%)	17 (4.3%)	
	Overseas	10 (2.5%)	4 (1.0%)		–	–	–		> 10 years	77 (19.4%)	15 (3.8%)	
To formally review a patient’s therapy and to make necessary changes to help promote positive health outcomes	B/M Pharm	71 (17.9%)	16 (4.0%)	0.031	State funded	169 (42.7%)	30 (7.6%)	0.031	< 5 years	65 (16.4%)	3 (0.8%)	0.009
	Pharm.D	268 (67.7%)	27 (6.8%)		Private	181 (45.7%)	16 (4.0%)		5–10 years	211 (53.3%)	25 (6.3%)	
	Overseas	11 (2.8%)	3 (0.8%)		–	–	–		> 10 years	74 (18.7%)	18 (4.5%)	
To supervise repeat prescriptions for patients according to agreed protocols	B/M Pharm	71 (17.9%)	16 (4.0%)	0.000*	State funded	172 (43.4%)	27 (6.8%)	0.035	< 5 years	65 (16.4%)	3 (0.8%)	0.001
	Pharm.D	275 (69.5)	20 (5.1%)		Private	183 (46.2%)	14 (3.5%)		5–10 years	217 (54.8%)	19 (4.8%)	
	Overseas	9 (2.3%)	5 (1.3%)		–	–	–		> 10 years	73 (18.4%)	19 (4.8%)	
To make dose adjustments to a patient’s medicine using protocols established with prescribers	B/M Pharm	71 (17.9%)	16 (4.0%)	0.000*	State funded	175 (44.2%)	24 (6.1%)	0.062	< 5 years	64 (16.2%)	4 (1.0%)	0.000*
	Pharm.D	278 (70.2%)	17 (4.3%)		Private	184 (46.5%)	13 (3.3%)		5–10 years	222 (56.1%)	14 (3.5%)	
	Overseas	10 (2.5%)	4 (1.0%)		–	–	–		> 10 years	73 (18.4%)	19 (4.8%)	
To prescribe therapy for a patient following a clinician’s diagnosis (partnership or supplementary prescribing)	B/M Pharm	79 (19.9%)	8 (2.0%)	0.001	State funded	187 (47.2%)	12 (3.0%)	0.816	< 5 years	66 (16.7%)	2 (0.5%0	0.032
	Pharm.D	282 (71.2)	13 (3.3%)		Private	184 (46.5%)	13 (3.3%)		5–10 years	224 (56.6%)	12 (3.0%)	
	Overseas	10 (2/5%)	4 (1.0%)		–	–	–		> 10 years	81 (20.5%)	11 (2.8%)	
To prescribe therapy for a patient independent of clinician’s diagnosis following an initial patient assessment (independent prescribing)	B/M Pharm	55 (13.9%)	32 (8.1%)	0.017	State funded	156 (39.4%)	43 (10.9%)	0.093	< 5 years	61 (15.4%)	7 (1.8%)	0.004
	Pharm.D	231 (58.7%)	64 (16.2%)		Private	140 (35.4%)	57 (14.4%)		5–10 years	173 (43.7%)	63 (15.9%)	
	Overseas	10 (2.5%)	4 (1.0%)		–	–	–		> 10 years	62 (15.7%)	30 (7.6%)	

* = significant *p*-value with Cramer V

**Table 3:** Experience of pharmacists with doctors (*N* = 396)

Experience	Education	Agree	Disagree	***P***-Value	Type	Agree	Disagree	***P***-Value	Experience	Agree	Disagree	***P***-Value
Pharmacists are a reliable source of general medicines information (i.e., specific facts about medicines, which can be found in standard references)	B/M Pharm	70 (17.7%)	17 (4.3%)	0.000*	State funded	180 (45.5%)	19 (4.8%)	0.016	< 5 years	68 (17.2%)	0 (0%)	0.000*
	Pharm.D	286 (72.2%)	9 (2.3%)		Private	190 (48%)	7 (1.8%)		5–10 years	224 (56.6%)	12 (3.0%)	
	Overseas	14 (3.5%)	0 (0%)		–	–	–		> 10 years	78 (19.7%)	14 (3.5%)	
Pharmacists routinely counsel patients regarding the safe and appropriate use of medicines	B/M Pharm	72 (18.2%)	15 (3.8%)	0.002	State funded	181 (45.7%)	18 (4.5%)	0.606	< 5 years	64 (16.2%)	4 (1.0%)	0.007
	Pharm.D	277 (69.9%)	18 (4.5%)		Private	182 (46%)	15 (3.8%)		5–10 years	222 (56.1%)	14 (3.5%)	
	Overseas	14 (3.5%)	0 (0%)		–	–	–		> 10 years	77 (19.4%)	15 (3.8%)	
Pharmacists routinely inform clinicians about the cost-effectiveness of therapy and give accurate advice regarding alternatives treatments	B/M Pharm	55 (13.9%)	32 (8.1%)	0.000*	State funded	171 (43.2%)	28 (7.1%)	0.005	< 5 years	63 (15.9%)	5 (1.3%)	0.004
	Pharm.D	249 (62.9%)	46 (11.6%)		Private	147 (37.1%)	50 (12.6%)		5–10 years	189 (47.7%)	47 (11.9%)	
	Overseas	14 (3.5%)	0 (0%)		–	–	–		> 10 years	66 (16.7%)	26 (6.6%)	
Pharmacists are willing to take personal responsibility for resolving any medicines-related problems they discover	B/M Pharm	73 (18.4%)	14 (3.5%)	0.000*	State funded	180 (45.5%)	19 (4.8%)	0.008	< 5 years	67 (16.9%)	1 (0.3%)	0.001
	Pharm.D	284 (71.7%)	11 (2.8%)		Private	191 (48.2%)	6 (1.5%)		5–10 years	225 (56.8%)	11 (2.8%)	
	Overseas	14 (3.5%)	0 (0%)		–	–	–		> 10 years	79 (19.9%	13 (3.3%)	
Pharmacists routinely inform clinicians if they discover clinical problems with prescriptions	B/M Pharm	78 (19.7%)	9 (2.3%)	0.005	State funded	193 (48.7%)	6 (1.5%)	0.000*	< 5 years	67 (16.9%)	1 (0.3%)	0.031
	Pharm.D	269 (67.9%)	26 (6.6%)		Private	163 (41.2%)	34 (8.6%)		5–10 years	209 (52.8%)	27 (6.8%)	
	Overseas	9 (2.3%)	5 (1.3%)		–	–	–		> 10 years	80 (20.2%)	12 (3.0%)	
Pharmacists frequently ask to clarify therapeutic objectives clinicians have for patients	B/M Pharm	56 (14.1%)	31 (7.8%)	0.000*	State funded	169 (42.7%)	30 (7.6%)	0.047	< 5 years	64 (16.2%)	4 (1.0%)	0.000*
	Pharm.D	279 (70.5%)	16 (4.0%)		Private	180 (45.5%)	17 (4.3%)		5–10 years	220 (55.6%)	16 (4.0%)	
	Overseas	14 (3.5%)	0 (0%)		–	–	–		> 10 years	65 (16.4%)	27 (6.8%)	
Pharmacists frequently let medics know that patients have experienced some problem with their medications	B/M Pharm	71 (17.9%)	16 (4.0%)	0.000*	State funded	172 (43.4%)	27 (6.8%)	0.035	< 5 years	65 (16.4%)	3 (0.8%)	0.001
	Pharm.D	275 (69.4%)	20 (5.1%)		Private	183 (46.2%)	14 (3.5%)		5–10 years	217 (54.8%)	19 (4.8%)	
	Overseas	9 (2.3%)	5 (1.3%)		–	–	–		> 10 years	73 (18.4%)	19 (4.8%)	
Pharmacists are focused on ensuring the safety of patients with respect to the therapeutic use of medicines	B/M Pharm	70 (17.7%)	17 (4.3%)	0.014	State funded	184 (46.5%)	15 (3.8%)	0.023	< 5 years	66 (16.7%)	2 (0.5%)	0.008
	Pharm.D	270 (68.2%)	25 (6.3%)		Private	168 (42.4%)	29 (7.3%)		5–10 years	211 (53.3%)	25 (6.3%)	
	Overseas	12 (3.0%)	2 (0.5%)		–	–	–		> 10 years	75 (18.9%)	17 (4.3%)	
Pharmacists respect the autonomy of patients and act to promote the concept of concordance	B/M Pharm	73 (18.4%)	14 (3.5%)	0.000*	State funded	181 (45.7%)	18 (4.5%)	0.025	< 5 years	66 (16.7%)	2 (0.5%)	0.002
	Pharm.D	284 (71.7%)	11 (2.8%)		Private	190 (48%)	7 (1.8%)		5–10 years	226 (57.1%)	10 (2.5%)	
	Overseas	14 (3.5%)	0 (0%)		–	–	–		> 10 years	79 (19.9%)	13 (3.3%)	
Pharmacists are practicing as autonomous clinicians	B/M Pharm	77 (19.4%)	10 (2.5%)	0.005	State funded	173 (43.7%)	26 (6.6%)	0.075	< 5 years	65 (16.4%)	3 (0.8%)	0.021
	Pharm.D	269 (67.9%)	26 (6.6%)		Private	182 (46%)	15 (3.8%)		5–10 years	214 (54.0%)	22 (5.6%)	
	Overseas	9 (2.3%)	5 (1.3%)		–	–	–		> 10 years	76 (19.2%)	16 (4.0%)	

* *=* significant *p*-value with Cramer V

**Table 4:** Perception of pharmacists regarding their involvement in medicines management (*N* = 396)

Perceptions	Education	Agree	Disagree	***P***-Value	Type	Agree	Disagree	***P***-Value	Experience	Agree	Disagree	***P***-Value
Pharmacists should increase their involvement in medicines management	B/M Pharm	45 (11.4%)	42 (10.6%)	0.000*	State funded	135 (34.1%)	64 (16.2%)	0.028	< 5 years	54 (13.6%)	14 (3.5%)	0.000*
	Pharm.D	229 (57.8%)	66 (16.7%)		Private	153 (38.6%)	44 (11.1%)		5–10 years	182 (46.0%)	54 (13.6%)	
	Overseas	14 (3.5%)	0 (0%)		–	–	–		> 10 years	52 (13.1%)	40 (10.1%)	
Current state or private funding does not support collaborative work between pharmacists and clinicians in medicines management	B/M Pharm	75 (18.9%)	12 (3.0%)	0.004	State funded	181 (45.7%)	18 (4.5%)	0.025	< 5 years	60 (15.2%)	8 (2.0%)	0.004
	Pharm.D	282 (71.2%)	13 (3.3%)		Private	190 (48%)	7 (1.8%)		5–10 years	229 (57.8%)	7 (1.8%)	
	Overseas	14 (3.5%)	0 (0%)		–	–	–		> 10 years	82 (20.7%)	10 (2.5%)	
Other than dispensing prescriptions, pharmacists are on the periphery of the core healthcare team	B/M Pharm	69 (17.4%)	18 (4.5%)	0.000*	State funded	189 (47.7%)	10 (2.5%)	0.804	< 5 years	67 (16.9%)	1 (0.3%)	0.000*
	Pharm.D	292 (73.7%)	3 (0.8%)		Private	186 (47%)	11 (2.8%)		5–10 years	230 (58.1%)	6 (1.5%)	
	Overseas	14 (3.5%)	0 (0%)		–	–	–		> 10 years	78 (19.7%)	14 (3.5%)	
Clinicians do not want pharmacists to provide medicines management services	B/M Pharm	63 (15.9%)	24 (6.1%)	0.000*	State funded	163 (41.2%)	36 (9.1%)	0.006	< 5 years	58 (14.6%)	10 (2.5%)	0.000*
	Pharm.D	273 (68.9%)	22 (5.6%)		Private	180 (45.5%)	17 (4.3%)		5–10 years	217 (54.8%)	19 (4.8%)	
	Overseas	7 (1.8%)	7 (1.8%)		–	–	–		> 10 years	68 (17.2%)	24 (6.1%)	
Patients would not subscribe to enhanced pharmacy practice services	B/M Pharm	38 (9.6%)	49 (12.4%)	0.000*	State funded	156 (39.4%)	43 (10.9%)	0.002	< 5 years	66 (16.7%)	2 (0.5%)	0.000*
	Pharm.D	281 (71.0%)	14 (3.5%)		Private	177 (44.7%)	20 (5.1%)		5–10 years	219 (55.3%)	17 (4.3%)	
	Overseas	14 (3.5%)	0 (0%)		–	–	–		> 10 years	48 (12.1%)	44 (11.1%)	
Medicines management by implication calls the clinician’s judgment into question	B/M Pharm	87 (22%)	0 (0%)	0.000*	State funded	181 (45.7%)	18 (4.5%)	0.031	< 5 years	59 (14.9%)	9 (2.3%)	0.021
	Pharm.D	250 (63.1%)	45 (11.4%)		Private	165 (41.7%)	32 (8.1%)		5–10 years	199 (50.2%)	37 (9.3%)	
	Overseas	9 (2.3%)	5 (1.3%)		–	–	–		> 10 years	88 (22.2%)	4 (1.0%)	
Medicines management challenges the clinician’s authority	B/M Pharm	60 (15.2%)	27 (6.8%)	0.051	State funded	152 (38.4%)	47 (11.9%)	0.189	< 5 years	46 (11.6%)	22 (5.6%)	0.478
	Pharm.D	217 (54.8%)	78 (19.7%)		Private	139 (35.1%)	58 (14.6%)		5–10 years	177 (44.7%)	59 (14.9%)	
	Overseas	14 (3.5%)	0 (0%)		–	–	–		> 10 years	68 (17.2%)	24 (6.1%)	
This enhanced clinical practice de-skills the clinicians/ practitioners	B/M Pharm	48 (12.1%)	39 (9.8%)	0.000*	State funded	154 (38.9%)	45 (11.4%)	0.000	< 5 years	62 (15.7%)	6 (1.5%)	0.000*
	Pharm.D	272 (68.7%)	23 (5.8%)		Private	180 (45.5%)	17 (4.3%)		5–10 years	215 (54.3%)	21 (5.3%)	
	Overseas	14 (3.5%)	0 (0%)		–	–	–		> 10 years	57 (14.4%)	35 (8.8%)	
I do not have time to discuss patient-related medicine issues with clinicians	B/M Pharm	87 (22%)	0 (0%)	0.000*	State funded	157 (39.6%)	42 (10.6%)	0.004	< 5 years	55 (13.9%)	13 (3.3%)	0.000*
	Pharm.D	232 (58.6%)	63 (15.9%)		Private	176 (44.4%)	21 (5.3%)		5–10 years	187 (47.2%)	49 (12.4%)	
	Overseas	14 (3.5%)	0 (0%)		–	–	–		> 10 years	91 (23.0%)	1 (0.3%)	
I feel inadequately trained to deal with clinicians on clinical medicine-related issues on behalf of patients	B/M Pharm	87 (22%)	0 (0%)	0.000*	State funded	193 (48.7%)	6 (1.5%)	0.000*	< 5 years	59 (14.9%)	9 (2.3%)	0.055
	Pharm.D	245 (61.9%)	50 (12.6%)		Private	153 (38.6%)	44 (11.1%)		5–10 years	200 (50.5%)	36 (9.1%)	
	Overseas	14 (3.5%)	0 (0%)		–	–	–		> 10 years	87 (22.0%)	5 (1.3%)	
I have sufficient confidence in my clinical knowledge to provide this service	B/M Pharm	87 (22.0%)	0 (0%)	0.000*	State funded	191 (48.2%)	8 (2.0%)	0.000*	< 5 years	50 (12.6%)	18 (4.5%)	0.000*
	Pharm.D	208 (52.5)	87 (22%)		Private	118 (29.8%)	79 (19.9%)		5–10 years	170 (42.9%)	66 (16.7%)	
	Overseas	14 (3.5%)	0 (0%)		–	–	–		> 10 years	89 (22.5%)	3 (0.8%)	
Patients will get conflicting information regarding medicines use if pharmacists develop their medicines management services	B/M Pharm	39 (9.8%)	48 (12.1%)	0.000*	State funded	77 (19.4%)	122 (30.8%)	0.585	< 5 years	19 (4.8%)	49 (12.4%)	0.009
	Pharm.D	95 (24%)	200 (50.5%)		Private	71 (17.9%)	126 (31.8%)		5–10 years	83 (21.0%)	153 (38.6%)	
	Overseas	14 (3.5%)	0 (0%)		–	–	–		> 10 years	46 (11.6%)	46 (11.6%)	
Enhanced clinical input will further develop my current relationship with clinicians	B/M Pharm	51 (12.9%)	36 (9.1%)	0.000*	State funded	162 (40.9%)	37 (9.3%)	0.001	< 5 years	66 (16.7%)	2 (0.5%)	0.000*
	Pharm.D	280 (70.7%)	15 (3.8%)		Private	183 (46.2%)	14 (3.5%)		5–10 years	219 (55.3%)	17 (4.3%)	
	Overseas	14 (3.5%)	0 (0%)		–	–	–		> 10 years	60 (15.2%)	32 (8.1%)	

* *=* significant *p*-value with Cramer V

## Discussion

Pharmaceutical care is an integral part of pharmacy practice in any healthcare setting, and its application varies from one country to another depending upon health regulations. The pharmaceutical care services are advanced in developed countries and involve pharmacists in more clinical and patient-oriented roles. However, the involvement of pharmacists in pharmaceutical care service and the extent of service coverage is limited in developing countries such as Pakistan as there are less pharmacists employed in hospitals. Their duties at most times, are confined to drug dispensing, procurement, and inventory management services, i.e., traditional pharmacy services [33–39]. It has been mentioned earlier that greater involvement of pharmacist in direct patient care and extensive interaction of pharmacists with allied health members would set the platform for improved pharmaceutical care services thereby benefiting the patients. This could only be achieved when pharmacists are involved in traditional duties to a lesser extent. The traditional duties could be performed by pharmacy technicians [10].

Most pharmacists mentioned that their role was clinically oriented and interacted with doctors on a daily basis. This response was significantly associated with workplace as pharmacists working in state funded hospitals interacted more often with doctors. This occurrence could be attributed to the change in healthcare policy that envisioned such role for pharmacists. This occurrence further highlights that Pakistani pharmacists now have a better understanding of pharmaceutical care and consider themselves as member of allied health team. Besides, they regard their work as clinical and patient-oriented.

Most pharmacists agreed that their role and duties in the primary health practice is of clinical nature. This indicates that they were aware of their role as a healthcare professional and, considered participation in drug prescribing and therapeutic procedures as must. This finding was linked to their education status as graduates with PharmD degree or overseas degree in pharmacy, as opposed to BPharm, had better acceptance towards these tasks. This finding could be attributed to the introduction of PharmD degree with clinical pharmacy courses. It is in line with the concept of enhancing pharmacists’ capabilities and allowing them to contribute to the primary healthcare system is being promoted, especially in developing countries [39, 40]. Henceforth, the introduction of PharmD degree has led to a positive change in pharmacists working in Pakistani healthcare settings. Pharmacists with a PharmD degree were observed to be better acquainted with their clinical responsibilities. Moreover, this finding is also in line with the role envisioned by the WHO for pharmacists, that is, to serve in an advisory capacity for other healthcare professionals in ensuring safe and appropriate use of medicines [14].

The data pertaining to the experience of pharmacists with the doctors highlighted that pharmacists were willing to collaborate with doctors to discuss patients’ condition and medication therapy. This occurrence was linked to education status and workplace of pharmacists as pharmacists with PharmD and working in state funded healthcare facilities, were exercising these tasks more often. However, the general perception of doctors was negative. Available evidence indicates that doctors perceived that pharmacists were incapable of providing direct healthcare service to patient [38–40]. This negative perception is still prevalent among doctors despite considering pharmacists as knowledgeable and experts in counseling patients about drug dosage and its safe use [24]. Besides, most patients in Pakistan are unaware that they can consult pharmacists if they experience any drug related problem during therapy [22, 24]. Due to this negative perception among doctors and public incognizance, the pharmacists have limited opportunities to assume the role of a direct patient care provider and mainly resort to practising managerial and administrative tasks in public and private healthcare sectors.

It was observed that the perception of pharmacists about their role in medicine management was quite positive. The pharmacists believed that they were capable of providing this service however, number of pharmacists who had such expertise and training was low. This occurrence is logical given the previous pharmacy curriculum that was focused on educating and training pharmacy graduates in drug manufacturing and dispensing. Thus, pharmacy graduates were mostly inclined towards pharmaceutical manufacturing and medicines dispensing. In India, a neighboring country, the situation was similar to Pakistan’s pharmacy education scenario as Indian pharmacy graduates and practicing pharmacists, despite being in a large number, were educated in preparation and dispensing of medicine only [37].

Pakistan’s pharmacy curriculum was revised in 2004; the 4 year degree of Bachelor of Pharmacy (B.Pharm) was upgraded to a full-time 5 year Doctor of Pharmacy (Pharm.D) degree program. It was upgraded to strengthen pharmacy graduates’ clinical knowledge and practice [37–42]. Studies mentioned that on an average 2587 pharmacy students graduated from Pakistan’s pharmacy schools every year. However, this number is not sufficient to meet the demands of pharmacy profession in country’s healthcare system [40]. According to available evidence, currently, 8102–10,000 pharmacists are working in Pakistan, of which 55% are involved in the production of pharmaceuticals, and only 15% are engaged in community pharmacy [23, 40]. Moreover, in addition to inadequate and untrained resource, doctors were reluctant to engage the pharmacists in medicine prescribing and related tasks [40, 41].

There is a need of understanding the role of pharmacists in Pakistani healthcare system and focus on their training and utilization in the health sector. The issue of under-recognized role could be addressed by attaching pharmacy schools with hospitals similar to medical schools so that pharmacy students may be able to practice their clinical knowledge in patient care and improve their clinical skills [37–41]. Moreover, PharmD graduates must be given extensive duties in the clinical pharmacy services so that they can progress and play their role as healthcare professionals effectively [43].

Apart from traditional pharmacy roles, pharmacists must assume the clinical role that contemporary healthcare service demands. Khan and colleagues highlighted that most doctors in Pakistan do not consider pharmacist as an integral member of allied healthcare team [42, 43]. There is a need to increase their awareness regarding the role pharmacists could play in Pakistan’s healthcare system. Evidence highlights that pharmacist’s inclusion in disease management as member of allied health team have improved patients’ treatment outcomes [44]. Randomized controlled trials involving pharmacist – driven pharmaceutical care model in Pakistani patients with chronic illnesses such as diabetes, hypertension, rheumatoid arthritis, etc., have shown significant improvements in treatment outcomes [44–47]. This evidence base would improve pharmacists’ standing in healthcare. In addition, allowing clinical pharmacists to accompany allied health members during clinical rounds and participation of pharmacists in continuous medical education activities alongside other healthcare professionals would be beneficial for recognition of pharmacists as a clinical member of allied health team. Additionally, spreading awareness about pharmacist’s role through regional and national pharmacy societies would also highlight pharmacists and their responsibilities among health professionals and public.

Furthermore, pharmacists would have to increase patient awareness about their role. They would have to perform their role as disease educator and counsellor. The patients would not be able to understand the impact a pharmacist could make in healthcare system unless the pharmacists take initiative. Patient education and counselling may not be clinically effective if the pharmacists do not have pharmaceutical care skills [22]. Therefore, it is essential to teach these skills to pharmacy students in PharmD curriculum and provide them with opportunities to practice during summer attachments or experiential training [22, 39]. This would be helpful as the pharmacy students would assume the role of pharmacists in future. There is a need to add provision of a counselling room in regulations for healthcare settings as this would provide opportunity for dissemination of information regarding disease and therapy. Moreover, such provision would enhance patient satisfaction and improve the recognition of pharmacists as a patient care provider [22, 39, 42, 43].

### Limitations of the study

The results of this study may be interpreted with caution as pharmacist from primary care hospitals, and community pharmacies were not included. Therefore, the findings of this study reflect the opinion of pharmacists from clinical settings only. The views may differ among pharmacist based on workplace. The study was a survey-based research and involved close-ended questions. Qualitative studies could investigate extensively and may provide in-depth explanation of expectations as well as experiences of pharmacists within the healthcare system of Pakistan.

## Conclusion

Pharmacists in Pakistan are willing to perform their duties and provide direct patient care using their clinical knowledge and expertise, pharmaceutical care skills and, experience for medicines management. It was observed that the updated curriculum improved their clinical knowledge and health policy regulations have provided more opportunities of pharmacists to collaborate with doctors in patient care. However, they seemed sceptical of performing advanced clinical pharmacy roles such as intervening in prescriptions and medication therapy, consultations, prescribing, etc. This scepticism was mainly due to a prevalent negative perception about pharmacists’ clinical capabilities among most doctors.

It is essential to integrate pharmacists’ clinical rotations with doctors to inculcate a professional relationship. Moreover, it would be helpful if training and seminars are conducted on the importance of clinical pharmacy services in Pakistan’s healthcare system. Such activities would provide an opportunity to recognize the accomplishments as well as identify limitations of pharmacists’ clinical role. Further studies are recommended to explore the concerns of pharmacists regarding these services so that they can be adequately addressed.

## Supplementary information

**Additional file 1.**

## Data Availability

The datasets used and/or analysed during the current study available from the corresponding author on reasonable request.

## Abbreviations

SPSS: Statistical Package for Social Sciences
USA: United States of America
Pharm.D: Doctor of Pharmacy
KPK: Khyber Pakhtunkhwa
AJK: Azad Jammu & Kashmir
WHO: World Health Organization
ACCP: American College of Clinical Pharmacy
