# Supplementary material for: Pharmacists' viewpoint towards their professional role in healthcare system: a survey of hospital settings of Pakistan
Source: BMC Health Serv Res. 2020 Jul 2;20:610. doi: 10.1186/s12913-020-05459-0 (PMC7330985; doi:10.1186/s12913-020-05459-0)
Supplement: Supplementary file 1 — Additional file 1. [file 12913_2020_5459_MOESM1_ESM.docx]

**Pharmacists’ perception of their role in Health Care**

**(A) Personal Information**

| **Age (years)** | **Gender** | **Professional Education** | **Place of Work** |
| --- | --- | --- | --- |
| _______ | Male | B. Pharm/ M. Pharm | State Funded Hospital |
|  | Female | Pharm.D | Private Hospital |
|  |  | Overseas Qualification | Other_____________ |
| **Current Job Title** | **Area of Practice** | **Are you a registered with Pharmacy Council?** | **Date of registration as a pharmacist** |
| ____________ | _________________ | Yes | _____________________ |
|  |  | No |  |
| **State** |  | Any other association____________ |  |
| Sindh |  |  | **As a Pharmacist, how long you have been**  **seeing Patients?** |
| Punjab |  |  |  |
| Baluchistan |  |  | ____________ |
| KPK |  |  |  |
| Capital Territory |  |  |  |
| AJK |  |  |  |
| Fata/ Gilgit |  |  |  |

**(B) Interactions with Clinicians**

| **How often do you work directly with Clinicians?** | **What are the most common reasons for these interactions? (tick multiple)** |
| --- | --- |
| Never/rarely | Drug availability queries |
| Once a Week | Drug alternative queries |
| Once a day | Drug dosage queries |
|  | Side effects queries |
|  | Drug interaction queries |
|  | Other, specify__________________ |
| **How would you categories pharmacy?** | **Which of the following you think best describes the Pharmacist?** |
| Professional | Clinician |
| Business | Technician |
| Both |  |

**C) Role of Pharmacists in Pakistan Healthcare**

As a Pharmacist in Pakistan’s Healthcare system, what are your expectations of your role/function?

[Indicate with a tick whether or not you agree or disagree with the following statements]

| **Expectation** | | **Agree** | **Disagree** |
| --- | --- | --- | --- |
| **A** | To educate patients and careers about the safe and appropriate use of medicines |  |  |
| **B** | To monitor and report patients’ responses to drug therapy |  |  |
| **C** | To be available for clinician consultation during ward rounds |  |  |
| **D** | To communicate or liaise with other healthcare professionals delivering patient care to facilitate positive health outcomes |  |  |
| **E** | To collaborate with other healthcare professionals as part of a multidisciplinary team |  |  |
| **F** | To provide advice to patients about their medication and/or health conditions |  |  |
| **G** | To dispense and accuracy check the supply of medicines to patient (counting pills, labeling, and accuracy checking) |  |  |
| **H** | To provide a “closed shop” service: receiving prescriptions from a practitioner and couriers the dispensed medicine to a patient only |  |  |
| **I** | To check that prescriptions are written for the correct dose for the patient |  |  |
| **J** | To check that prescriptions do not have drug-drug interactions |  |  |
| **K** | To check that a prescription is not contraindicated for the patient |  |  |
| **L** | To advise clinicians and others about the cost-effectiveness of medicines |  |  |
| **M** | To formally review a patient’s therapy and to make necessary changes to help promote positive health outcomes |  |  |
| **N** | To supervise repeat prescriptions for patients according to agreed protocols |  |  |
| **O** | To make dose adjustments to a patient’s medicine using protocols established with prescribers |  |  |
| **P** | To prescribe therapy for a patient following a clinician’s diagnosis (partnership or supplementary prescribing) |  |  |
| **Q** | To prescribe therapy for a patient independent of clinician’s diagnosis following an initial patient assessment (independent prescribing) |  |  |
| **R** | Any other comments: | | |

**D)** **Pharmacist Experience with Healthcare Professionals**

What are your views of your peers?

[Indicate with a tick whether or not you agree or disagree with the following statements]

| **Experiences** | | **Agree** | **Disagree** |
| --- | --- | --- | --- |
| **A** | Pharmacists are a reliable source of general medicines information (i.e., specific facts about medicines, which can be found in standard references) |  |  |
| **B** | Pharmacists routinely counsel patients regarding the safe and appropriate use of medicines |  |  |
| **C** | Pharmacists routinely inform clinicians about the cost-effectiveness of therapy and give accurate advice regarding alternatives treatments |  |  |
| **D** | Pharmacists are willing to take personal responsibility for resolving any medicines-related problems they discover |  |  |
| **E** | Pharmacists routinely inform clinicians if they discover clinical problems with prescriptions |  |  |
| **F** | Pharmacists frequently ask to clarify therapeutic objectives clinicians have for patients |  |  |
| **G** | Pharmacists frequently let medics know that patients have experienced some problem with their medications |  |  |
| **H** | Pharmacists are focused on ensuring the safety of patients with respect to the therapeutic use of medicines |  |  |
| **I** | Pharmacists respect the autonomy of patients and act to promote the concept of concordance |  |  |
| **J** | Pharmacists are practicing as autonomous clinicians |  |  |
| **K** | Any other comments: | | |

**E) Pharmacists’ Perception of the Undergraduate Curriculum**

Are you satisfied with the current curriculum of Pharmacy Education in Pakistan?

[Indicate with a tick whether or not you agree or disagree with the following statements]

| **Perception Regarding Curriculum** | | **Agree** | **Disagree** |
| --- | --- | --- | --- |
| **A** | The curriculum adequately addresses all the aspects of contemporary Pharmacy practice |  |  |
| **B** | The curriculum adequately addresses all clinical aspects of Pharmacy practice |  |  |
| **C** | Generally, I am satisfied with the Pharmacy undergraduate curriculum |  |  |
| **D** | The balance of theoretical and practical aspects of the curriculum is correct for contemporary practice |  |  |
| **E** | The introduction of a 5-year Pharm-D program in Pakistan’s pharmacy schools will develop better practitioners |  |  |
| **F** | Any other comments: | | |

**F) Pharmacist involvement in Medicines Management**

What you think of the following?

| **Medicines Management Services** | | **Yes** | **No** |
| --- | --- | --- | --- |
| **A** | Pharmacists should increase their involvement in medicines management |  |  |
| **B** | Current state or private funding does not support collaborative work between pharmacists and clinicians in medicines management |  |  |
| **C** | Other than dispensing prescriptions, pharmacists are on the periphery of the core healthcare team |  |  |
| **D** | Clinicians do not want me to provide medicines management services |  |  |
| **E** | Patients would not subscribe to enhanced pharmacy practice services |  |  |
| **F** | Medicines management by implication calls the clinician’s judgment into question |  |  |
| **G** | Medicines management challenges the clinician’s authority |  |  |
| **H** | This enhanced clinical practice de-skills the clinicians/ practitioners |  |  |
| **I** | I don’t have time to discuss patient-related medicine issues with clinicians |  |  |
| **J** | I feel inadequately trained to deal with clinicians on clinical medicine-related issues on behalf of patients |  |  |
| **K** | I have sufficient confidence in my clinical knowledge to provide this service |  |  |
| **L** | Patients will get conflicting information regarding medicines use if pharmacists develop their medicines management services |  |  |
| **M** | Enhanced clinical input will further develop my current relationship with clinicians |  |  |
| **N** | Any other comments: | | |
